# 5′ preS1 Mutations To Prevent Large Envelope Protein Expression from Hepatitis B Virus Genotype A or Genotype D Markedly Increase Polymerase-Envelope Fusion Protein

**DOI:** 10.1128/jvi.01723-21

**Published:** 2022-03-09

**Authors:** Jing Zhang, Quan Yuan, Yongxiang Wang, Yuzhou Wang, Wenqing Yuan, Ningshao Xia, Yumei Wen, Jisu Li, Shuping Tong

**Affiliations:** a Department of Pathobiology, Key Laboratory of Medical Molecular Virology, School of Basic Medical Sciences, Fudan University, Shanghai, China; b State Key Laboratory of Molecular Vaccinology and Molecular Diagnostics, School of Public Health, Xiamen University, Xiamen, China; c Liver Research Center, Rhode Island Hospital, The Warren Alpert School of Medicine, Brown University, Providence, Rhode Island, USA; University of Southern California

**Keywords:** hepatitis B virus, large envelope protein, polymerase-envelope fusion protein, preS1 region, RNA splicing, 3.5-kb RNA, genotype

## Abstract

Hepatitis B virus (HBV) large (L) envelope protein is translated from 2.4-kb RNA. It contains preS1, preS2, and S domains and is detected in Western blotting as p39 and gp42. The 3.5-kb pregenomic RNA produces core and polymerase (P) proteins. We generated L-minus mutants of a genotype A clone and a genotype D clone from 1.1-mer or 1.3-mer construct, with the former overproducing pregenomic RNA. Surprisingly, mutating a preS1 ATG codon(s) or introducing a nonsense mutation soon afterwards switched secreted p39/gp42 to a p41/p44 doublet, with its amount further increased by a nonsense mutation in the core gene. A further-downstream preS1 nonsense mutation prevented p41/p44 production. Tunicamycin treatment confirmed p44 as the glycosylated form of p41. In this regard, splicing of 3.5-kb RNA to generate a junction at nucleotides (nt) 2447 to 2902 for genotype D enables translation of p43, with the N-terminal 47 residues of P protein fused to the C-terminal 371 residues of L protein. Indeed p41/p44 were detectable by an antibody against the N terminus of P protein and eliminated by a nonsense mutation at the 5′ P gene or a point mutation to prevent that splicing. Therefore, lost L (and core) protein expression from the 1.1-mer or 1.3-mer construct markedly increased p41/p44 (p43), the P-L fusion protein. Cotransfection with an expression construct for L/M proteins reversed high extracellular p41/p44 associated with L-minus mutants, suggesting that L protein retains p43 in wild-type HBV to promote its intracellular degradation. Considering that p43 lacks N-terminal preS1 sequence critical for receptor binding, its physiological significance during natural infection and therapeutic potential warrant further investigation.

**IMPORTANCE** The large (L) envelope protein of hepatitis B virus (HBV) is translated from 2.4-kb RNA and detected in Western blotting as p39 and gp42. Polymerase (P) protein is expressed at a low level from 3.5-kb RNA. The major spliced form of 3.5-kb RNA will produce a fusion protein between the first 47 residues of P protein and a short irrelevant sequence, although also at a low level. Another spliced form has the same P protein sequence fused to L protein missing its first 18 residues. We found that some point mutations to eliminate L and core protein expression from overlength HBV DNA constructs converted p39/gp42 to p41/gp44, which turned out to be the P-L fusion protein. Thus, the P-L fusion protein can be expressed at extremely high level when L protein expression is prevented. The underlying mechanism and functional significance of this variant form of L protein warrant further investigation.

## INTRODUCTION

The 3.2-kb circular DNA genome of hepatitis B virus (HBV) has four unidirectional genes in the order precore/core, polymerase (P), preS1/preS2/S (envelope), and X, with the P gene overlapping the 3′ precore/core, the entire envelope gene, and 5′ X gene. Four size forms of unspliced and coterminal mRNAs are transcribed from covalently closed circular DNA (cccDNA) in the nucleus to generate seven viral proteins (1). The longest transcript, 3.5 kb, driven by the core promoter, is terminally redundant and has a heterogeneous 5′ end. The longer version (precore RNA [pcRNA]) has intact precore region at its 5′ end to enable expression of precore/core protein, which is converted by proteolytic cleavage to hepatitis B e antigen (HBeAg). The slightly shorter pregenomic RNA (pgRNA) translates core protein from the 5′-most AUG codon or P protein from a downstream AUG. It also drives HBV genome replication through its packaging into nucleocapsids (core particles) assembled from core protein, where it is converted by the copackaged P protein into partially double-stranded DNA. The 27-nm nucleocapsids with such mature DNA genomes are enveloped and secreted as 42-nm virions.

The large (L), middle (M), and small (S) envelope proteins contain preS1/preS2/S, preS2/S, and S domains alone, respectively, through alternative translation initiation from in-frame ATG codons in the preS1, preS2, and S regions of the envelope gene (2, 3). L protein is translated from the 2.4-kb RNA, while M and S proteins are products of the 2.1-kb RNA. The preS1 domain has 119 amino acids (aa) for most HBV genotypes but 108 aa for genotype D, while the preS2 and S domains have 55 aa and 226 aa, respectively. The S domain has a facultative glycosylation site to produce two size forms of L (p39/gp42), M (gp33/gp36), and S (p24/gp27) proteins. L protein is myristoylated at its N-terminal glycine (the initiating methionine is cleaved off) (4, 5). It initiates virion morphogenesis by interacting with nucleocapsids through the matrix domain (6), while S protein, the most abundant HBV protein, drives virion secretion. The majority of S protein is rather secreted as 22-nm subviral particles (SVPs) lacking internal nucleocapsids. SVPs outnumber virions in the bloodstream in large excess and are detected as hepatitis B surface antigen (HBsAg) (7). L protein cannot be secreted if expressed without S protein, and it inhibits SVP production according to the L/S protein ratio (8–10). Although M protein is present on both virions and SVPs, mutating the preS2 ATG codon to prevent its expression does not eliminate virion or SVP production (11–15).

Cloning the 3.2-kb HBV genome into a vector will prevent transcription of the terminally redundant pcRNA and pgRNA. This can be overcome by converting the monomeric HBV DNA construct into a tandem dimer or multimer (16). Properly designed overlength (such as 1.3-mer) HBV genomes can also generate pcRNA and pgRNA from endogenous core promoter (17). Alternatively, inserting the DNA equivalent of pgRNA without a poly(A) tail into a vector downstream of a strong foreign promoter permits much higher levels of pgRNA transcription and genome replication (18). We recently used such a 1.1-mer construct of a genotype A clone and a genotype D clone to study the impact of S and core protein coexpression on steady-state levels of L and M proteins (19). Surprisingly, mutating the preS1 ATG codon(s) or introducing a premature nonsense mutation at the 5′ preS1 region failed to eliminate L protein expression but rather slightly increased its size.

## RESULTS

### Mutating a preS1 ATG codon(s) from a 1.1-mer construct failed to eliminate extracellular L protein from a genotype A or D clone but slightly increased its size.

**FIG 1:**
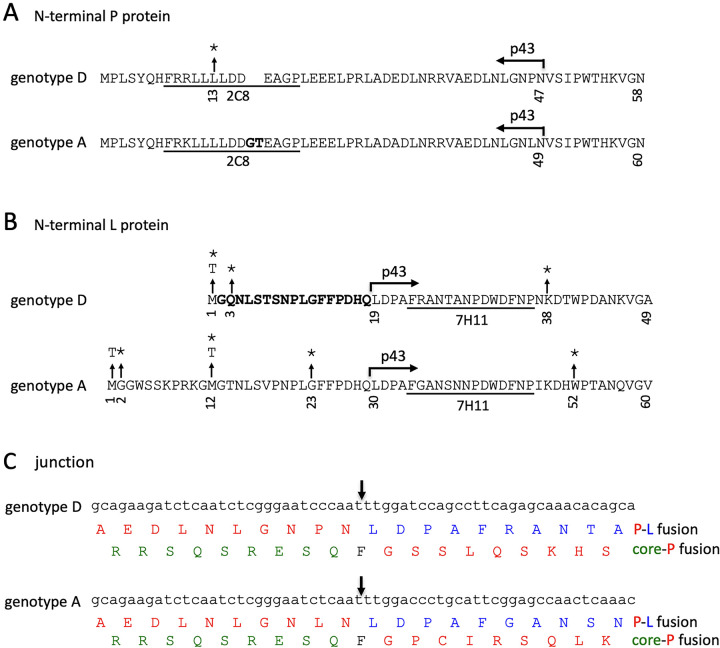
N-terminal sequences of HBV P and L proteins as well as the junction for p43, the P-L fusion protein. (A) N-terminal 58 aa of the P protein of geno1.2 (genotype D) and its counterpart in geno5.4 (genotype A). p43 contains the N-terminal 47 aa of P protein for genotype D but 49 aa for genotype A due to a 2-aa internal insertion (boldface). The epitope for 2C8, an anti-P MAb, is underlined. An L13* nonsense mutation was introduced into geno1.2 to abolish p43 expression. *, nonsense mutation. (B) The N-terminal 49 aa of the L protein for geno1.2 (genotype D) and its counterpart for geno5.4 (genotype A). Genotype A has a 33-nt insertion at the 5′ end of the preS1 region relative to genotype D to add 11 aa at the N terminus of the L protein. The size difference in the preS1 domain would be lost in p43, which starts at the 19th preS1 residue for genotype D but the 30th residue for genotype A. Boldface indicates preS1 aa 2 to 18 of genotype D, essential for binding to the high-affinity HBV receptor. Underlining indicates the epitope for 7H11, the anti-preS1 MAb used in the present study. Missense mutations to prevent translation initiation and nonsense mutations to prematurely terminate translation are shown. (C) RNA junction generated by splicing and fusion among P, envelope, and core genes. The junction, as indicated by an arrowhead, is nt 2447-nt 2902 for genotype D but nt 2453-nt 2941 for genotype A. That would fuse the 5′ P gene with 3′ envelope gene to generate p43 and the entire core gene (with mutated last codon) with the 3′ P gene (Table 2). Shown are the predicted amino acid sequences of the P-L fusion protein and core-P fusion protein for geno1.2 of genotype D and geno5.4 of genotype A. Red, blue, and green indicate amino acids encoded by the P, envelope, and core genes, respectively.

**FIG 2:**
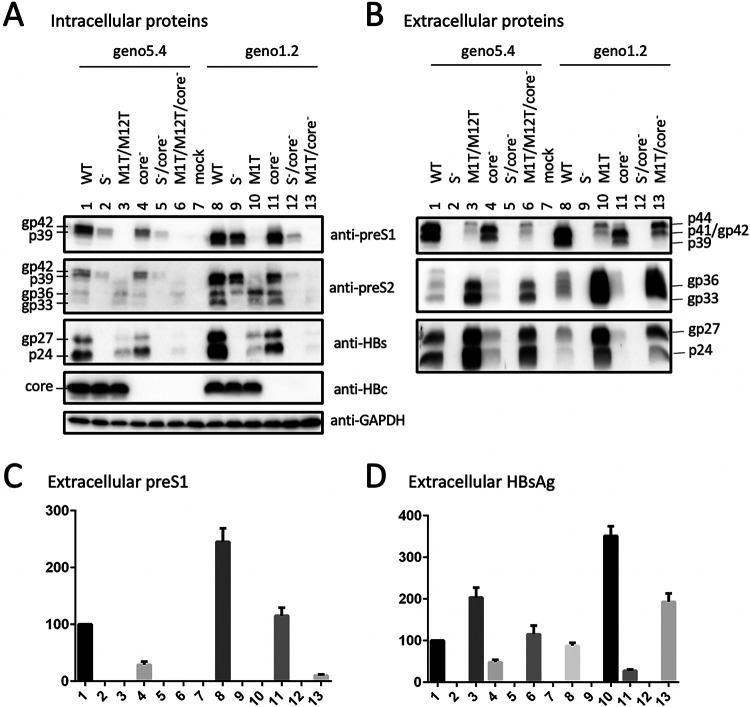
Mutating the preS1 ATG codon(s) to prevent L protein expression from geno5.4 or geno1.2 generated a variant form of L protein with slower mobility. Huh7 cells grown in 6-well plates were transfected with the parental 1.1-mer WT construct of geno5.4 (genotype A) or geno1.2 (genotype D), the corresponding core-minus, S-minus, or L-minus mutant (M1T/M12T for geno5.4 and M1T for geno1.2), or double mutants. (A and B) Western blot analysis of cell lysate (A) and culture supernatant (B) using the indicated antibodies, with prior PEG precipitation of SVPs, virions, and naked core particles from 120 μl of culture supernatant. The L (p39/gp42), M (gp33/gp36), S (p24/gp27), and core proteins were detected by the anti-preS1 (7H11), anti-preS2 (3E6), anti-S (Novus), and anti-HBc (2A7) antibodies, respectively. GAPDH (glyceraldehyde-3-phosphate dehydrogenase) served as a loading control for cell lysate. (C and D) ELISA for secreted preS1 antigen (C) and HBsAg (D), with values for WT geno5.4 set at 100.

**FIG 3:**
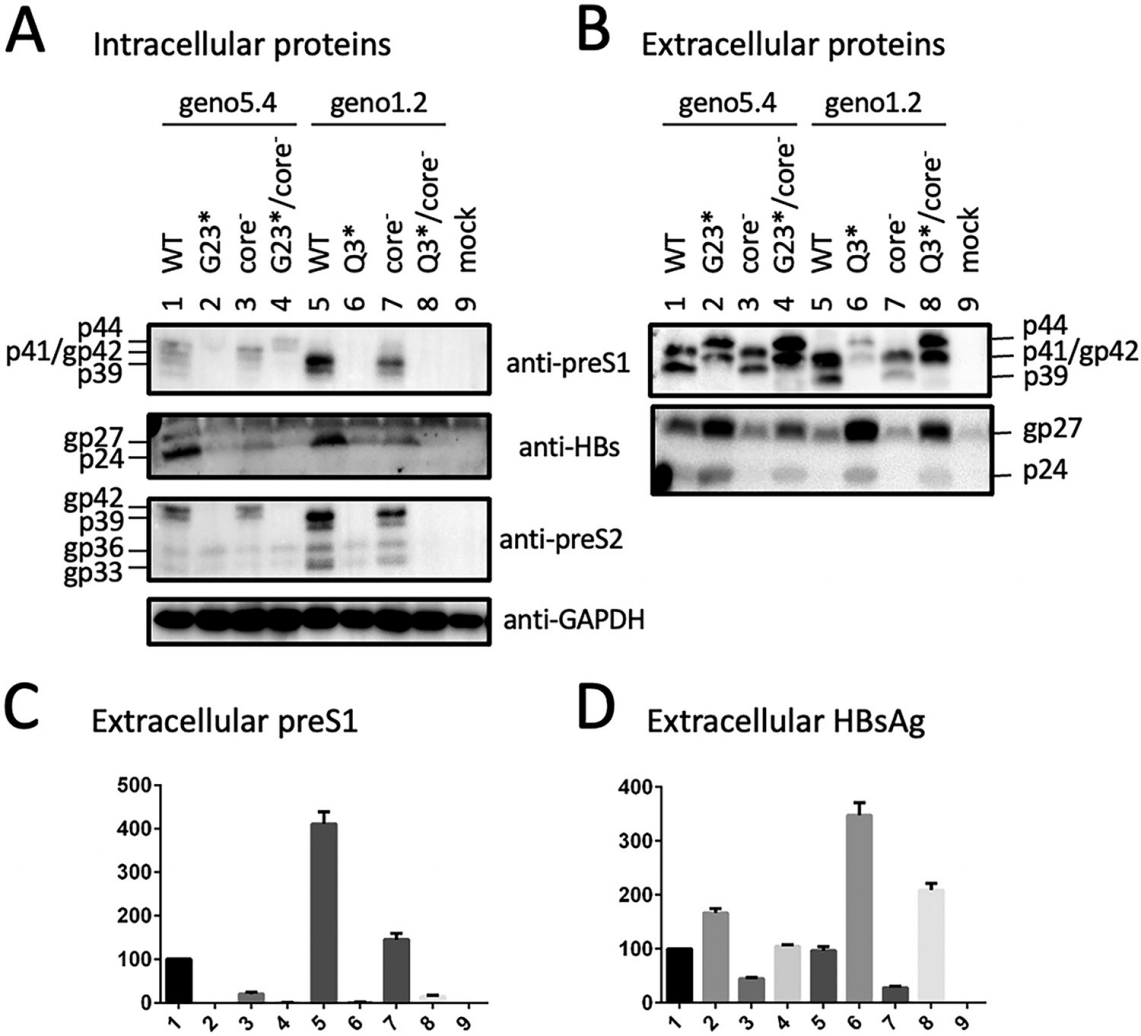
The preS1 nonsense mutation G23* in genotype A and Q3* in genotype D could also generate the variant form of L protein. Huh7 cells were transfected with L-minus mutant G23* of geno5.4 and Q3* of geno1.2, alone or together with the core-minus mutation. The WT construct and core-minus mutant served as controls. (A and B) Western blot analysis of envelope proteins in cell lysate (A) and culture supernatant (after PEG precipitation) (B). (C and D) ELISA detection of secreted preS1 antigen (C) and HBsAg (D) from culture supernatant, with values for WT geno5.4 set at 100.

We recently generated a 1.1-mer construct for geno5.4, a genotype A clone, and geno1.2, a genotype D clone (19, 20). Since genotype A has an extra 33 nucleotides (nt) at the 5′ preS1 region to add 11 aa to L protein (Fig. 1B), geno5.4 produced a slightly slower-migrating p39/gp42 doublet than geno1.2 in transiently transfected Huh7 cells (Fig. 2A and B, top, lanes 1 and 4 versus 8 and 11). 7H11, the anti-preS1 MAb used for L protein detection (21, 22), recognizes aa 23 to 36 in genotype D, corresponding to aa 34 to 47 in genotype A (Fig. 1B). An L-minus mutant of geno1.2 was generated by mutating its preS1 ATG codon to ACG (M1T), which was silent in the overlapping P gene (Table 1). Since genotype A contains two in-frame ATG codons in the preS1 region separated by 33 nt, both were mutated to ACG (M1T/M12T). Surprisingly a variant form of L protein (doublet of 41 and 44 kDa) replaced p39/gp42 in culture supernatant for both geno5.4 and geno1.2 (Fig. 2B, top, lanes 3 and 10). The new bands were of similar mobility between the two genotypes, with its 41-kDa band migrating faster than gp42 of genotype A but similarly to gp42 of genotype D (compare lanes 3 to 6 in the top panel of Fig. 3B). In contrast, abolishing S protein expression prevented L protein secretion without generating p41/p44 (Fig. 2B, top, lanes 2 and 9). While abolishing core protein expression via the C48* nonsense mutation reduced extracellular L, M, and S proteins for the parental construct as expected (Fig. 2B, compare lanes 1 and 4 and lanes 8 and 11) (19), it rather increased the p41/p44 doublet (but not M or S protein) from the L-minus mutants (compare lanes 3 and 6 and lanes 10 and 13). Enzyme-linked immunosorbent assay (ELISA) of culture supernatant using a commercial kit revealed lost preS1 signal for both the M1T mutant of geno1.2 and the M1T/M12T mutant of geno5.4, although adding the core-minus mutation generated a weak signal for geno1.2 (Fig. 2C, lanes 3, 6, 10, and 13). Lost L protein expression increased secretion of both M and S proteins, leading to higher HBsAg titers (Fig. 2B and D, compare lanes 1 and 3 and lanes 8 and 10).

**TABLE 1:** Mutations in the 1.1-mer construct to prevent or alter L, S, core, or P protein expression or 3.5-kb RNA splicing from the genotype A or D clone^*a*^

Genotype	Protein or splicing	Change (nt)	Change in aa or splicing	Change (aa, overlapping gene)
A	L	T2855C/ T2888C	M1T/M12T	P: silent
A	L	G2857T	G2*	P: G184V
A	L	A2887T/T2888A	M12*	P: H194L
A	L	G2920T	G23*	P: G205V
A	L	G3008A	W52*	P: silent
D	L	T2849C	M1T	P: silent
D	L	A2848T/T2849A	M1*	P: H181L
D	L	C2854T	Q3*	P: A183V
D	L	A2959T	K38*	P: Q218L
A, D	S	T156C	M1T	P: silent
A	Core	C2044A	C48*	NA
D	Core	T2044A	C48*	NA
D	P	T2344A	L13*	Core: silent
D	Splicing of 3.5-kb RNA	A2900C	Loss of 3.0-kb spliced RNA	P: silent

a Genotype A clone, geno5.4; genotype D clone, geno1.2. NA, not applicable.

### The p41/p44 doublet could also be generated by introducing the preS1 nonsense mutation Q3* into genotype D and G23* but not G2* into genotype A.

**FIG 4:**
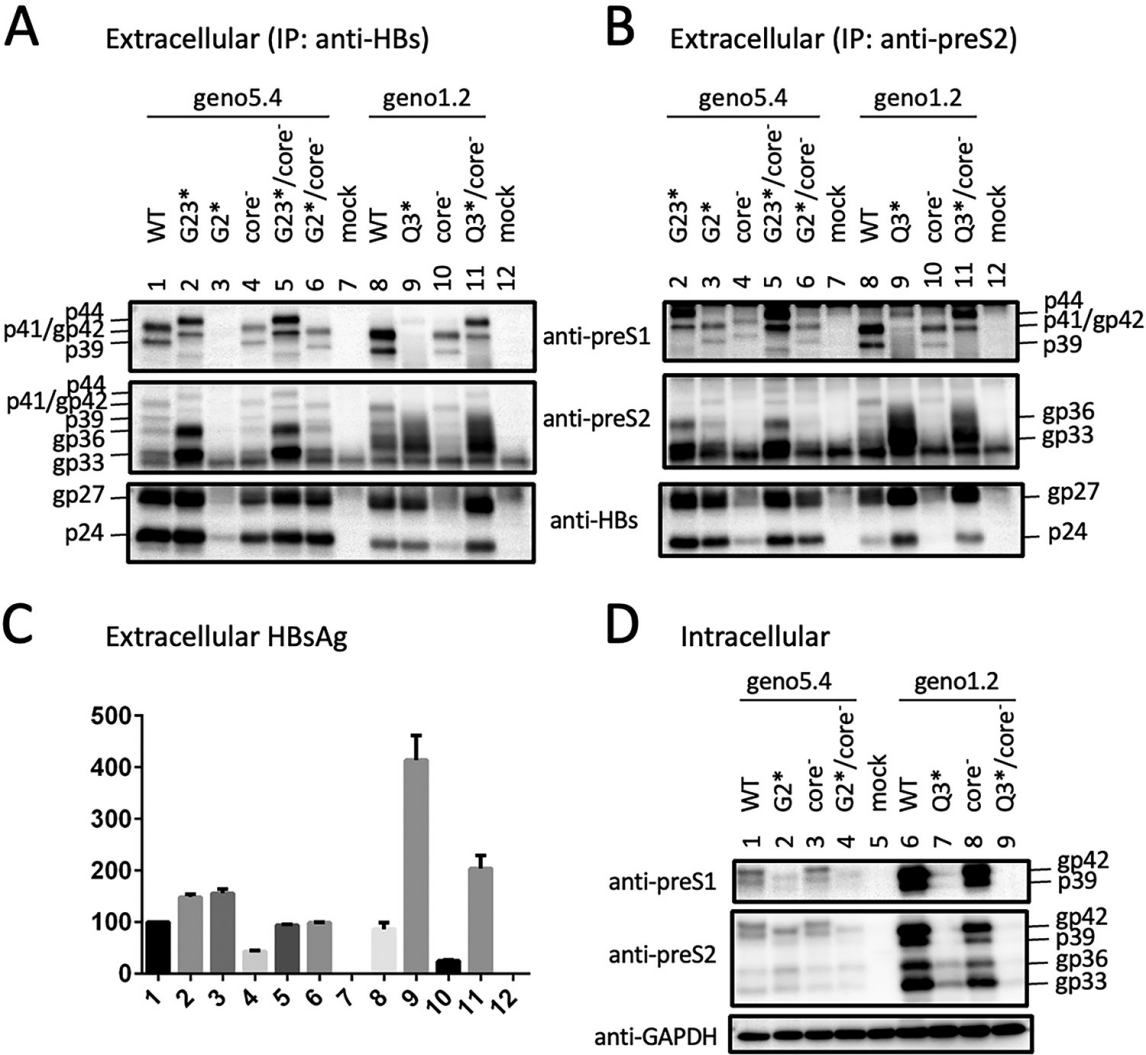
Impact of a G2* nonsense mutation in genotype A on L protein expression and IP-Western blot analysis to further characterize the variant form of L protein. The preS1 nonsense mutation G2* or G23* was introduced into genotype A clone geno5.4, while the Q3* mutation was introduced into genotype D clone geno1.2. The WT construct and nonsense mutants, alone or together with the core-minus mutation, were transfected into Huh7 cells seeded in 6-well plates. Panels A to C represent one set of transfection experiments. (A and B) Western blot analysis of secreted L, M, and S proteins following immunoprecipitation of 250 μl culture supernatant with an anti-HBs antibody (Abcam) (A) or the anti-preS2 antibody (B). (C) ELISA for secreted HBsAg, with the value for WT geno5.4 set at 100. (D) Western blot analysis of intracellular L and M proteins from another transfection experiment involving the G2* mutant of geno5.4.

Considering that protein translation could sometimes be initiated by a non-ATG codon (such as ACG, used in this study) (23–25), in another approach we eliminated L protein expression by converting preS1 codon 3 of geno1.2 from CAG to TAG (Q3*) and codon 23 of geno5.4 from GGA to TGA (G23*) (Table 1). Both nonsense mutations eliminated preS1 antigen in culture supernatant, although adding the core-minus mutation generated a low ELISA signal (Fig. 3C, lanes 2, 4, 6, and 8). However, Western blotting with 7H11 MAb revealed strong signals of a p41/p44 doublet for both Q3* and G23* mutants (Fig. 3B, top, lanes 2 and 6). Adding the core-minus mutation again enhanced the p41/p44 doublet in culture supernatant (Fig. 3B, top, lanes 4 and 8) and also made p41/p44 clearly visible in cell lysate for the G23* mutant (Fig. 3A, top, lane 4). On the other hand, a G2* mutation of geno5.4 shifted the gp42/p39 band to comigrate with L protein of genotype D (Fig. 4D, top, compare lanes 1, 2, and 6), suggesting that a nonsense mutation immediately downstream of the initiating ATG in genotype A caused translation reinitiation from the next in-frame ATG to generate shortened L protein like genotype D.

### The preS1 nonsense mutation W52* in genotype A and K38* in genotype D eliminated p41/p44 production.

**FIG 5:**
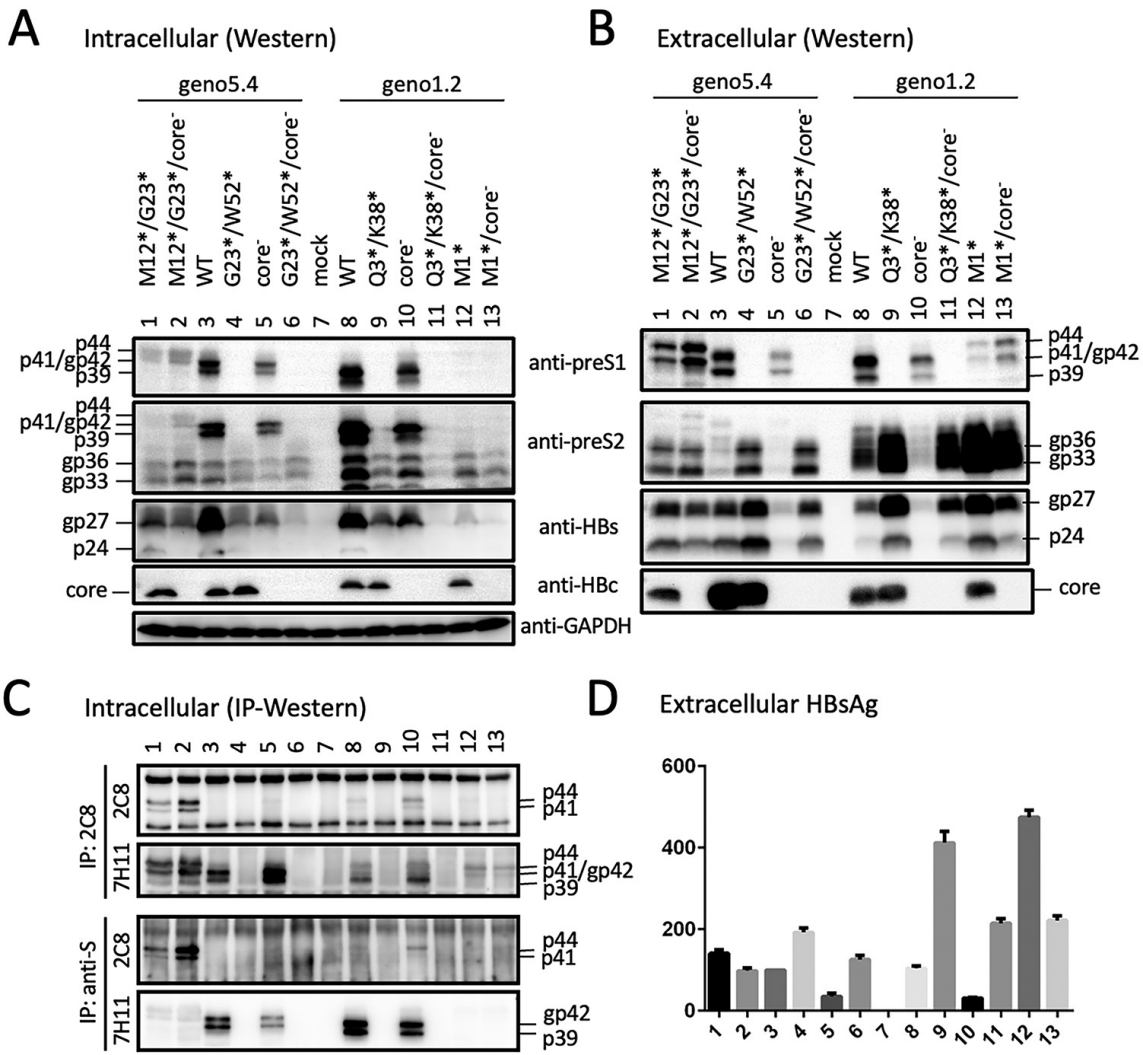
The combined preS1 nonsense mutations G23*/W52* in genotype A and Q3*/K38* in genotype D prevented p41/p44 production. The preS1 nonsense mutations M12*/G23* and G23*/W52* were introduced into geno5.4, while M1* and Q3*/K38* were introduced into geno1.2. Such mutants alone or together with core-minus mutation were transfected into Huh7 cells grown in 6-well plates using the WT construct as a control. (A and B) Direct Western blot analysis of envelope and core proteins from cell lysate (A) and PEG precipitate of culture supernatant (B). (C) IP-Western blot analysis of cell lysate. One-third of each cell lysate was subjected to IP with 2C8 (anti-P) MAb or anti-HBs (Abcam) antibody, followed by sequential Western blotting with 2C8 and 7H11 (anti-preS1) antibodies. (D) ELISA for secreted HBsAg, with the value for WT geno5.4 set at 100.

Intrigued by the above observations, we further generated an M12*/G23* double nonsense mutant of geno5.4 to ensure lost L protein expression. For geno1.2, an M1* (ATG-to-TAG) mutant was generated. However, both mutants continued to produce a p41/p44 doublet, which once again was intensified by the core-minus mutation (Fig. 5B, top, lanes 1, 2, 12, and 13). Nevertheless, adding a W52* mutation to the G23* mutant of geno5.4 eliminated p41/p44 species even for the core-minus mutant (Fig. 5B, top, lanes 4 and 6). The same effect was achieved by K38* mutation on top of the Q3* mutation in geno1.2 (Fig. 5B, top, lanes 9 and 11). In fact, the W52* mutation alone in geno5.4 and the K38* mutation alone in geno1.2 sufficed to prevent p41/p44 production (19).

### p41/p44 has a preS2 epitope.

We recently mapped the epitope for 3E6, an anti-preS2 monoclonal antibody (MAb) (26), to aa 13 to 22 in genotype D (J. Zhang, unpublished data). 3E6 could detect L protein in addition to M protein in cell lysate but was less efficient at detecting extracellular L protein for geno1.2 due to a much higher abundance of M protein (Fig. 2A and B, second panel). Consistent with a previous report (27), M protein secreted from genotype D migrated as a broad smear rather than distinct bands (Fig. 2B, second panel, compare lanes 3 and 10). This MAb confirmed generation of a genotype d-like L protein by the G2* mutation in geno5.4 (Fig. 4D, middle, compare lanes 1, 2, and 6). It detected a p41/p44 doublet from an M12*/G23*/core-minus mutant of geno5.4 in both cell lysate and culture supernatant (Fig. 5A and B, second panel, lane 2). In another approach, 3E6 was used to immunoprecipitate L and M proteins from culture supernatant. Subsequent Western blotting with 7H11 could detect the p41/p44 doublet from the Q3* mutant of geno1.2 and the G23* mutant of geno5.4 (Fig. 4B, top). Since S protein (p24/gp27) could also be detected from the immunoprecipitate (Fig. 4B, bottom), coimmunoprecipitation (co-IP) might pull down another envelope protein coanchored on the same SVPs. We also used a horse polyclonal anti-HBs antibody (Abcam) to immunoprecipitate the envelope proteins from culture supernatant, followed by Western blotting with 7H11 or 3E6. Here, 3E6 could detect the p41/p44 species from the G23* mutant of geno5.4 with or without a core-minus mutation and the Q3*/core-minus mutant of geno1.2 (Fig. 4A, middle, lanes 2, 5, and 11). Taken together, the results of Western blotting with MAb 3E6, either direct or following IP with anti-S antibody, revealed a preS2 epitope on p41/p44.

### p44 is the glycosylated form of p41.

**FIG 6:**
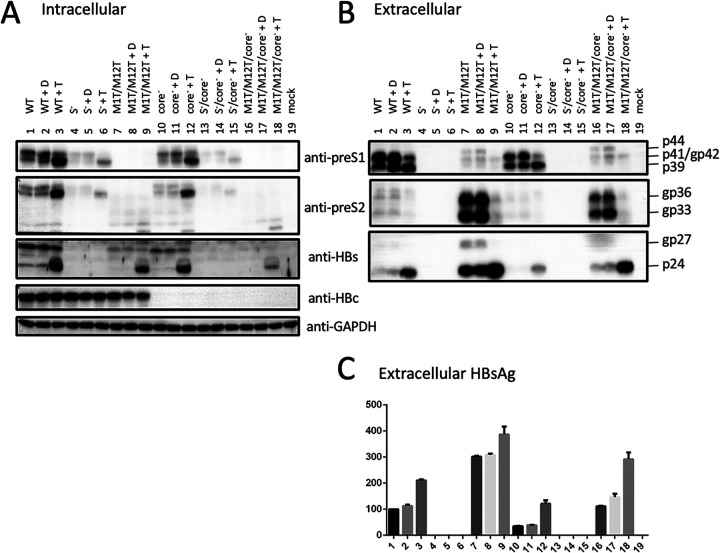
Impact of tunicamycin treatment on different size forms of L, M, and S proteins and p41/p44, the genotype A clone. Huh7 cells were transfected in triplicate with 6 gen5.4 constructs, including the L-minus (M1T/M12T) mutant and the L-minus core-minus double mutant. One well of cells was treated with tunicamycin dissolved in 1% DMSO (+T). The second well was cultured in medium containing same amount of DMSO (+D), while the third well was left untreated. Cells and culture supernatant were harvested 42 h later. (A) Western blot analysis of intracellular envelope and core proteins using GAPDH as a loading control. S protein was detected by rabbit polyclonal antibody (Novus). (B) Western blot analysis of secreted L, M, and S proteins following PEG precipitation of culture supernatant. S protein was detected with MAb 45E9. (C) ELISA for secreted HBsAg, with value for the WT construct without any treatment set at 100.

**FIG 7:**
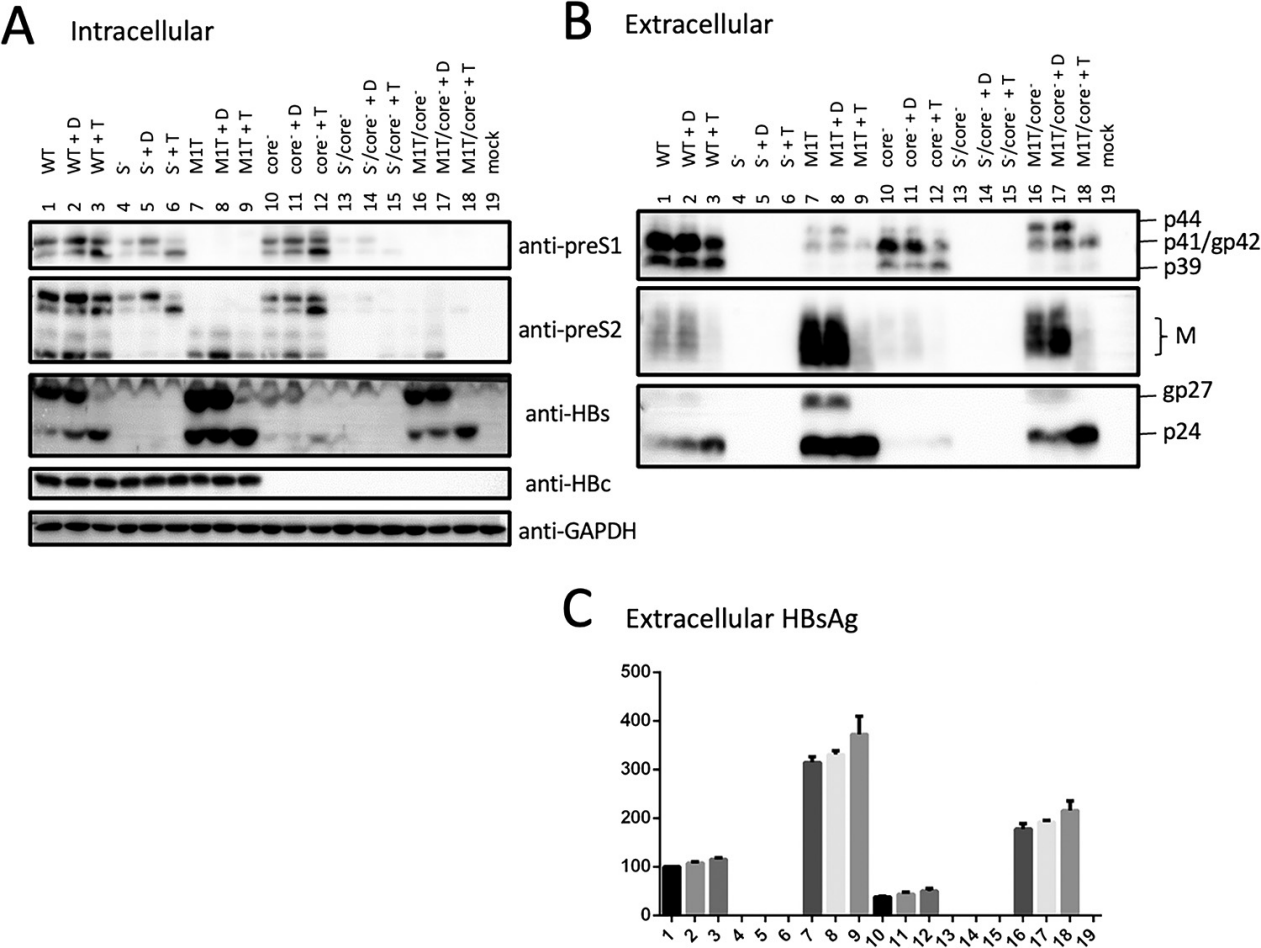
Impact of tunicamycin treatment on different size forms of L, M, S proteins and p41/p44, the genotype D clone. Huh7 cells were transfected in triplicate with 6 gen1.2 constructs, including those with the L-minus (M1T) mutation. Subsequently, one well was treated with tunicamycin dissolved in 1% DMSO (+T), another well was cultured in medium containing same amount of DMSO (+D), and the third well was left untreated. Cells and culture supernatant were harvested 42 h later. (A) Western blot analysis of intracellular envelope and core proteins using GAPDH as a loading control. S protein was detected with a rabbit polyclonal antibody (Novus). (B) Western blot analysis of secreted envelope proteins following PEG precipitation of culture supernatant. S protein was detected by 45E9 MAb. (C) ELISA for secreted HBsAg, with the value for the WT construct without tunicamycin or DMSO treatment set at 100.

If p41 and p44 were indeed variant forms of L protein, then p44 should be glycosylated p41. Huh7 cells transfected with a 1.1-mer construct of geno5.4 were treated with tunicamycin dissolved in dimethyl sulfoxide (DMSO), with untreated cells and cells treated with DMSO to serve as negative controls. For the 4 constructs capable of S protein expression, tunicamycin markedly increased nonglycosylated S protein (p24) in culture supernatant (Fig. 6B, bottom). M protein was poorly visible in cell lysate, but in culture supernatant, both gp33 and gp36 were markedly reduced by tunicamycin treatment (Fig. 6B, middle). In this regard, a previous work found that tunicamycin treatment converted intracellular M protein from gp33/gp36 into p30, which was poorly secreted (27). For all the 4 constructs capable of L protein expression, tunicamycin increased intracellular p39 at the expense of gp42 (Fig. 6A, top and second panels). In culture supernatant gp42 was much reduced relative to p39 for WT construct and its core-minus mutant (no L protein was secreted from S-minus mutants) (Fig. 6B, top, lanes 1 to 3 and 10 to 12). For the L-minus (M1T/M12T) mutant with or without a core-minus mutation, tunicamycin treatment eliminated p44 but not p41 in culture supernatant (Fig. 6B, top, lanes 7 to 9 and 16 to 18). For geno1.2, tunicamycin treatment also eliminated p44 but not p41 in culture supernatant from its L-minus (M1T) mutant with or without a core-minus mutation (Fig. 7B, top, lanes 7 to 9 and 16 to 18). Therefore, p44 is the glycosylated form of p41.

### Mutating the splicing acceptor site eliminated p41/p44 production from the genotype D clone.

**FIG 8:**
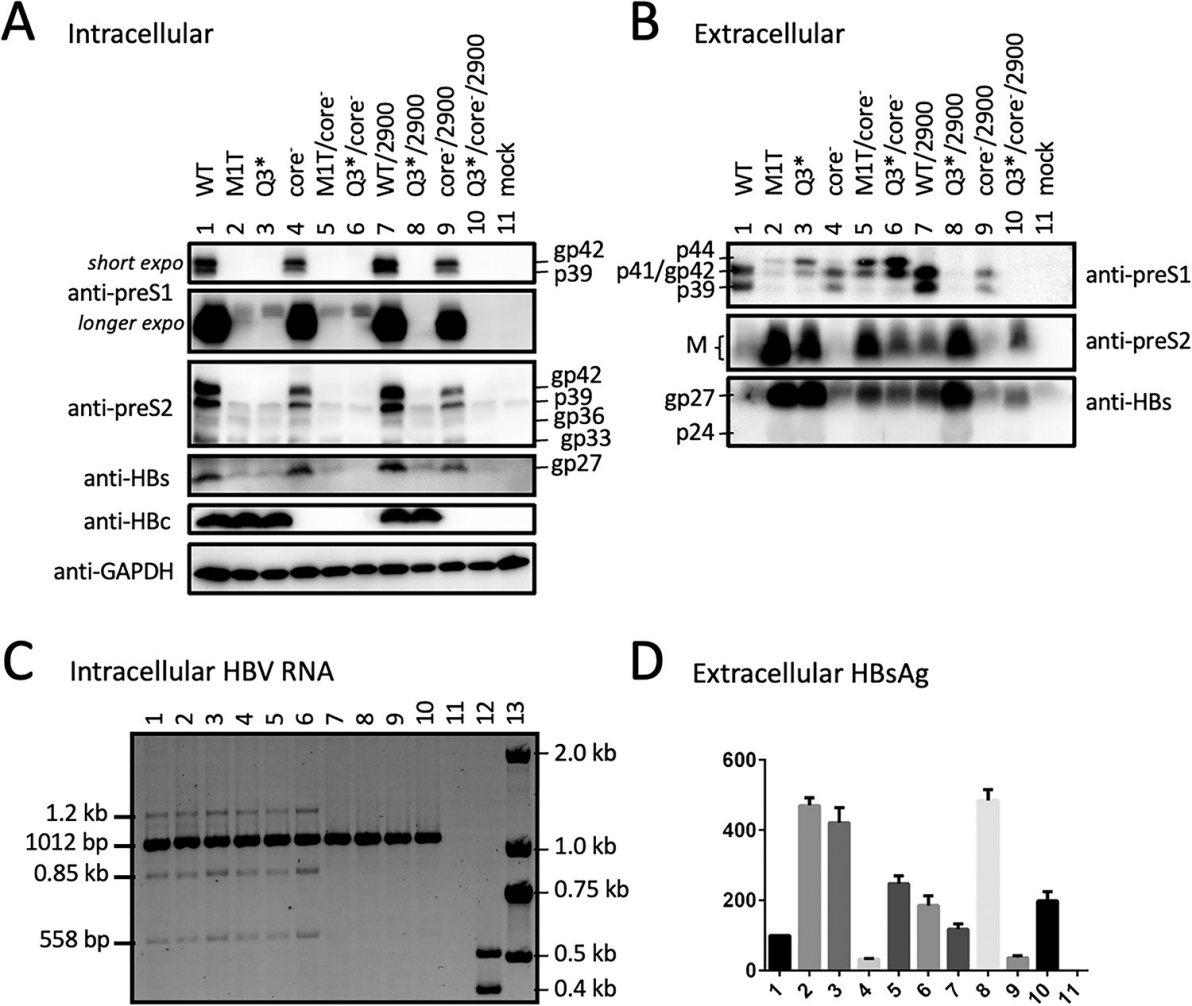
An A2900C mutation to prevent production of the 3.0-kb spliced RNA eliminated p41/p44 production from an L-minus mutant of genotype D. The A2900C mutation was introduced into the WT geno1.2 construct, its core-minus mutant, and the Q3* L-minus mutant with or without the core-minus mutation. Both the original constructs and A2900C mutants were transfected into Huh7 cells. (A) Western blot analysis of intracellular L, M, S, and core proteins using GAPDH as a loading control. (B) Western blot analysis of secreted L, M, and S proteins following PEG precipitation of culture supernatant. (C) RT-PCR analysis of the 3.5-kb RNA or its 3.0-kb singly spliced form in cell lysate. The expected size for the unspliced 3.5-kb pgRNA is 1,012 bp, while the 454-nt deletion to join nt 2447-nt 2902 should shorten the PCR product to 558 bp. Lane 11, negative control for PCR. Lanes 12 and 13, DNA size markers. (D) ELISA for secreted HBsAg, with the value for the WT construct set at 100.

Previous work using the P protein expression construct or 1.1-mer construct of a genotype D clone identified a 43-kDa P-envelope fusion protein (28, 29). p43 expression was attributed to single splicing of the 3.5-kb RNA to join nt 2447 to nt 2902 (29). Therefore, p43 of genotype D is a 418-aa protein with the first 18 aa of L protein replaced by the N-terminal 47 aa of P protein (Fig. 1A and B). p43 expression from their genotype D clone was abolished by an A2900C point mutation at the splicing acceptor site (29). While the point mutation did not impair p39/gp42 expression from the wild-type (WT) construct of geno1.2 (Fig. 8A and B, top, compare lanes 1 and 7), it abolished p41/p44 production from the Q3* L-minus mutant even in the presence of core-minus mutation (Fig. 8B, top, lanes 3, 6, 8, and 10). Reverse transcription-PCR (RT-PCR) of RNA extracted from transfected Huh7 cells using the primer pair nt2310-nt2329 and nt139-nt120 generated not only a 1.0-kb fragment corresponding to unspliced 3.5-kb RNA but also three minor bands of about 1.2 kb, 0.85 kb, and 0.56 kb (Fig. 8C). The 0.56-kb band was consistent with single splicing between nt 2447 and nt 2902. This band was present in the WT construct but not much increased in the presence of an M1T or Q3* mutation, even if accompanied by the core-minus mutation (Fig. 8C, compare lanes 1, 5, and 6). All four constructs with the A2900C point mutation lost the 0.56-kb band (Fig. 8C, lanes 7 to 10). Cloning and sequencing of HBV DNA fragments extracted from agarose gels confirmed unspliced HBV sequence for the 1.0-kb band and an nt 2447-nt 2902 junction for the 0.56-kb band. Most clones derived from the 1.2-kb band also had unspliced HBV sequence, while most clones from the 0.85-kb band had the nt 2447-nt 2902 junction. The reason for their aberrant migration was unclear.

### p41/p44 could be recognized by an antibody targeting the N terminus of P protein and was eliminated by a nonsense mutation at the 5′ P gene.

**FIG 9:**
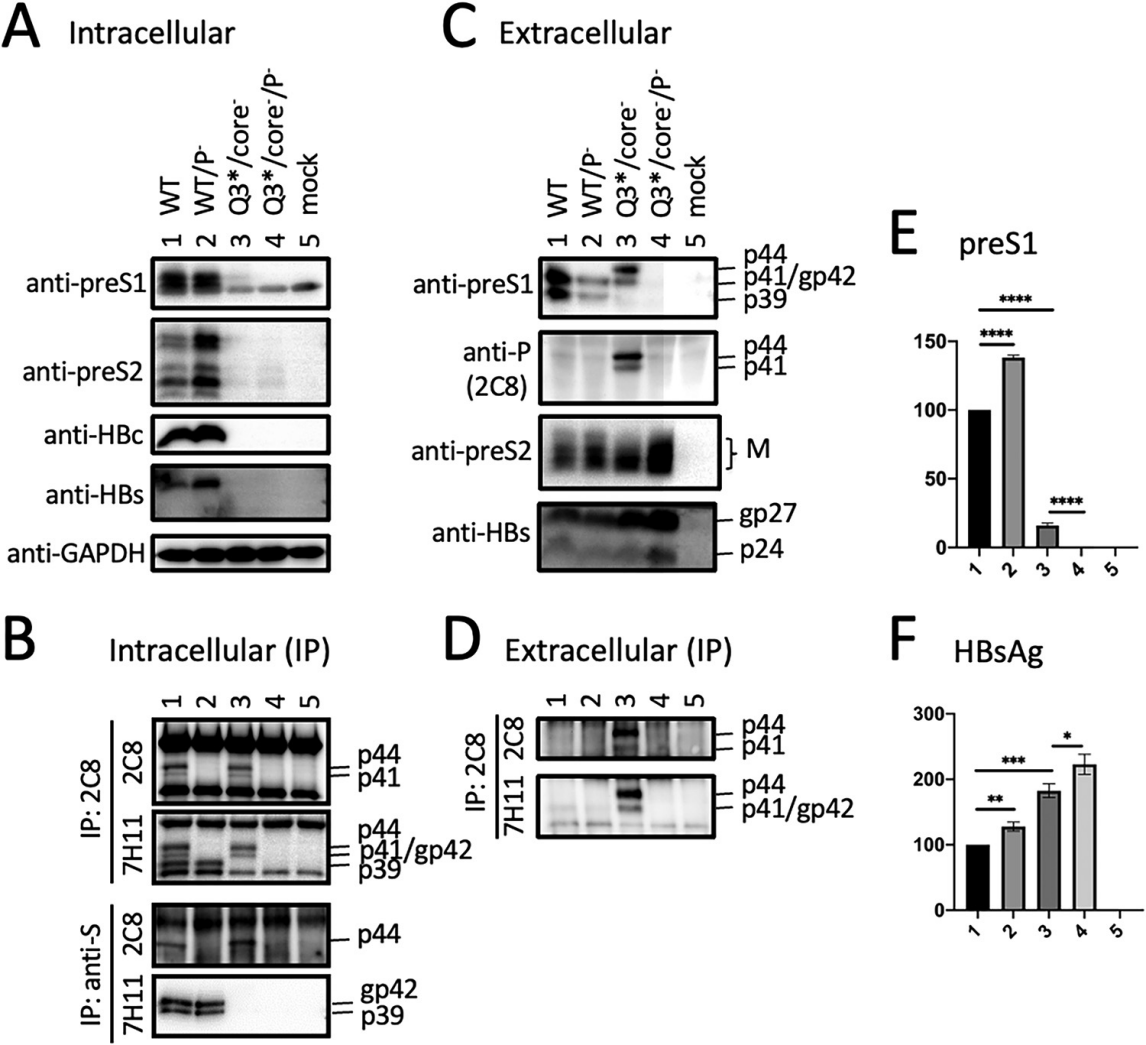
p41/p44 recognition by an anti-P antibody and its elimination by a nonsense mutation in the P gene. An L13* P-minus mutation was introduced into WT geno1.2 or its Q3*/core-minus double mutant. The parental constructs and P-minus mutants were transfected into Huh7 cells. (A and C) Direct Western blot analysis of cell lysate (A) and PEG precipitate of culture supernatant (C) with the indicated antibodies. 2C8 is an anti-P MAb. (B and D) IP-Western blot analysis of cell lysate (B) and culture supernatant (D). One-third of the cell lysate and 600 μl of culture supernatant were subjected to IP with either anti-P (2C8) or anti-HBs (Abcam) antibody, as indicated. (E and F) ELISA for secreted preS1 antigen (E) and HBsAg (F), with the value for WT geno1.2 set at 100. *, *P* < 0.05; **, *P* < 0.01; ***, *P* < 0.001; ****, *P* < 0.0001.

MAb 2C8, which recognizes aa 8 to 20 in HBV P protein, can work in Western blotting, IP, and immunofluorescent staining (30–32). In Western blotting, it detected p41/p44 but not p39/gp42 from culture supernatant (Fig. 9C, second panel, lanes 1 and 3). 2C8 also precipitated p41/p44 from cell lysate or culture supernatant, which could be revealed in subsequent Western blotting by either 2C8 itself or 7H11, the anti-preS1 MAb (Fig. 5C and 9B and D). An L13* nonsense mutation introduced into the 5′ P gene (Fig. 1A) did not impair L protein expression from the WT construct of geno1.2 but eliminated p41/p44 from the Q3*/core-minus mutant (Fig. 9C, top, lanes 2 and 4). Therefore, p41/p44 contains N-terminal P protein sequence, as expected for p43, the P-L fusion protein.

### Intracellular P-L fusion protein was unaltered by L-minus mutations in a 1.1-mer construct of geno1.2 but much increased by some L-minus mutations in geno5.4.

**FIG 10:**
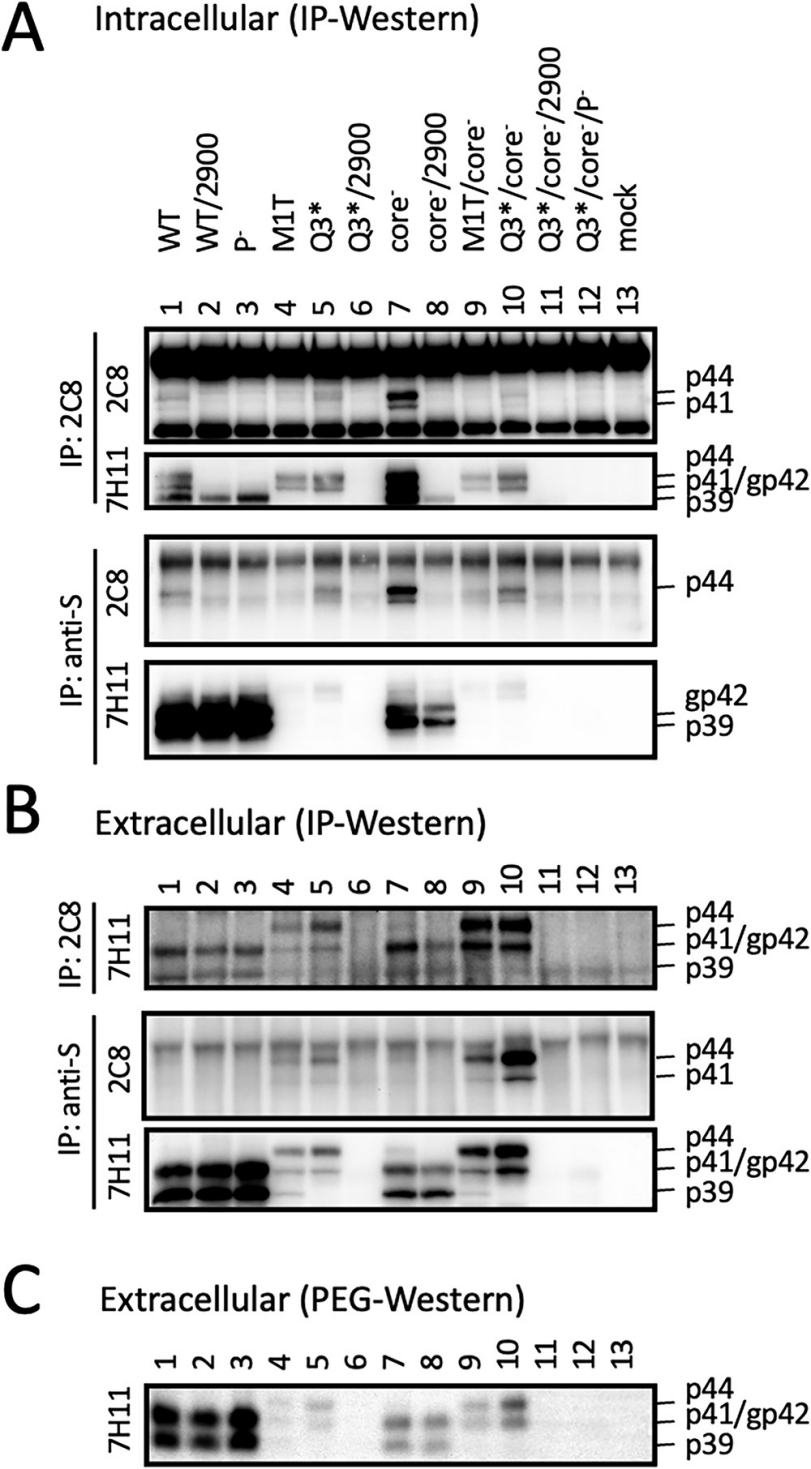
Impact of L-minus, core-minus, and P-minus mutations or a splicing site mutation in genotype D on intracellular and extracellular levels of P-L fusion protein. Huh7 cells seeded in 6-well plates were transfected with the 1.1-mer geno1.2 construct containing the core-minus, L-minus (M1T or Q3*), or P-minus (L13*) mutation or the A2900C splicing site mutation. (A and B) IP-Western blot analysis of intracellular (A) and secreted (B) L protein and P-L fusion protein. One-third of the cell lysate and 600 μl of culture supernatant were subjected to IP with the 2C8 anti-P antibody or anti-HBs (Abcam) antibody, followed by sequential Western blotting with anti-P and anti-preS1 (7H11) antibodies. (C) Western blot analysis of secreted L protein or P-L fusion protein using the anti-preS1 antibody. SVPs were PEG precipitated from 120 μl of culture supernatant.

Based on Western blots with the 7H11 MAb, p41/p44 was easily detected from culture supernatant of cells transfected with L-minus mutants of either geno5.4 or geno1.2. In contrast, intracellular p41/p44 was detectable only from cells transfected with G23*/core-minus mutant of geno5.4 or its M12*/G23* mutant with or without core-minus mutation (Fig. 3A and 5A, top panels). The large excess of gp42 from the WT construct made it difficult for the anti-preS1 antibody to reveal a low intracellular level of p41/p44 (Fig. 8A, second panel). Prior IP with the anti-P antibody markedly reduced L protein signal to increase the sensitivity and specificity of detection for the fusion protein. Using this approach, we found little intracellular p41/p44 from the WT construct of geno5.4 and validated its marked increase by the M12*/G23* L-minus mutation (Fig. 5C, top, lanes 1 to 3). For geno1.2, p41/p44 was already detectable in cell lysate from the WT construct but not much increased by L-minus mutations (Fig. 5C, 9B, and 10A, top panels). The L-minus mutations rather markedly increased p41/p44 in culture supernatant (Fig. 9D and 10B and C). The core-minus mutation primarily increased extracellular p41/p44 for the L-minus mutants (Fig. 10B, compare lanes 4 and 9 and lanes 5 and 10) but intracellular p41/p44 for the WT construct (Fig. 10A, compare lanes 1 and 7).

### p41/p44 could also be produced in HepG2 cells.

**FIG 11:**
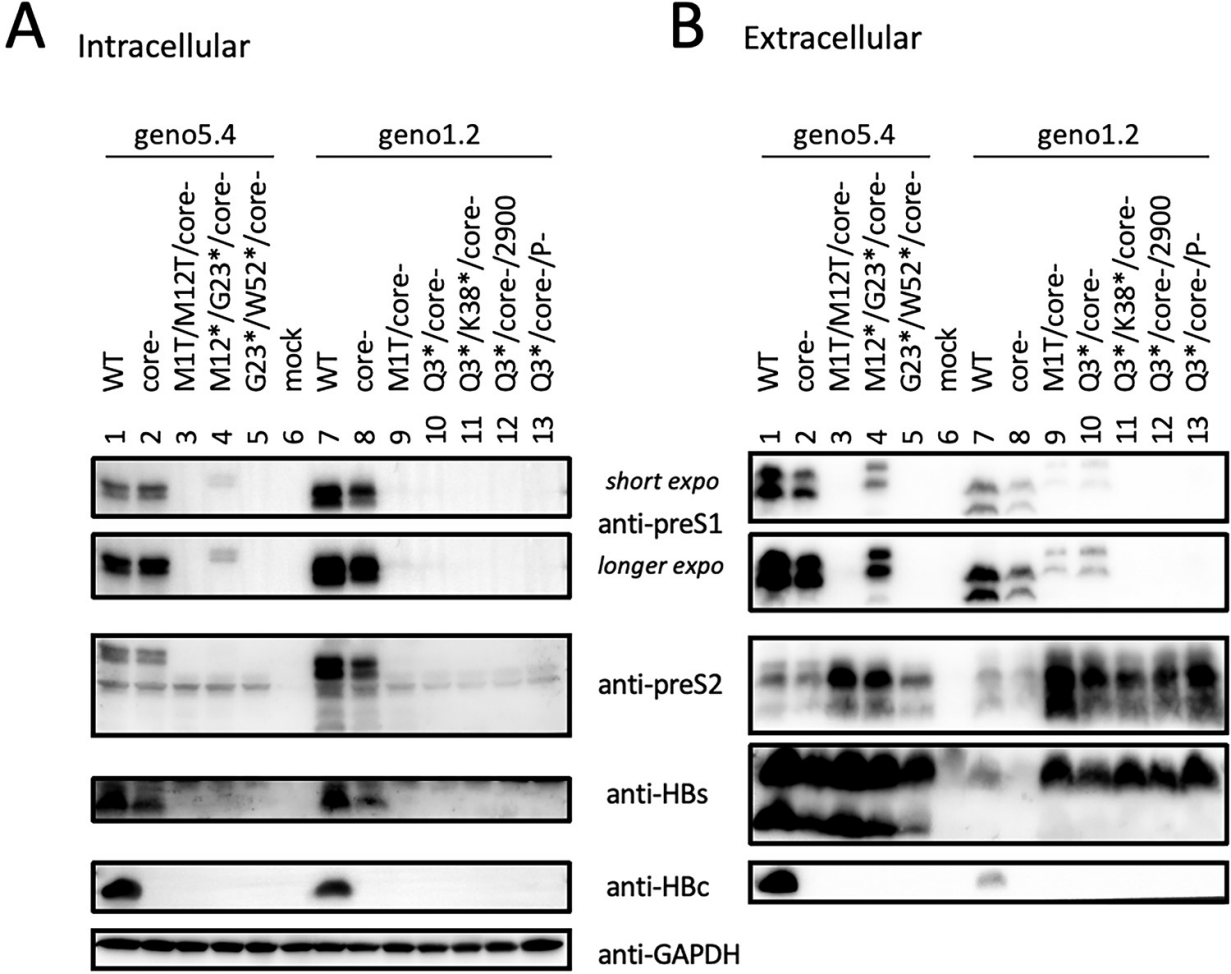
Production of p41/p44 from HepG2 cells transfected with 1.1-mer construct of some L-minus mutants of geno5.4 or geno1.2. HepG2 cells seeded in 6-well plates were transfected with 2 μg of indicated geno5.4 or geno1.2 constructs and harvested 4 days later. (A) Western blot analysis of cell lysate using anti-preS1 (7H11), anti-preS2 (3E6), anti-HBs (Novus), and anti-HBc (2A7) antibodies. GAPDH served as a loading control. (B) Western blot analysis of culture supernatant using the same set of anti-HBV antibodies following PEG precipitation of 200 μl of culture supernatant.

So far, all the findings regarding p41/p44 were based on transfection experiments in Huh7 cell line. HepG2, another human hepatoma cell line, supports not only HBV genome replication following transfection with the HBV genome but also infection with HBV particles following reconstitution with sodium taurocholate cotransporting polypeptide (NTCP) (33). We therefore transfected several geno5.4 and geno1.2 constructs into HepG2 cells. Extracellular p41/p44 was produced by the M1T/core-minus or Q3*/core-minus mutant of geno1.2 but not by its Q3*/K38*/core-minus, Q3*/core-minus/2900, or Q3*/core-minus/P-minus mutant (Fig. 11B, top two panels). The doublet could be detected from both cell lysate and culture supernatant of HepG2 cells transfected with a M12*/G23*/core-minus but not a G23*/W52*/core-minus mutant of geno5.4 (Fig. 11A and B, top two panels). In contrast to Huh7 cells, HepG2 cells failed to generate p41/p44 from the M1T/M12T/core-minus mutant of geno5.4. Whether this was attributed to residual L protein expression from the ACG codon(s) remains to be determined.

### p41/p44 could also be produced by the 1.3-mer HBV DNA construct without pgRNA overproduction.

**FIG 12:**
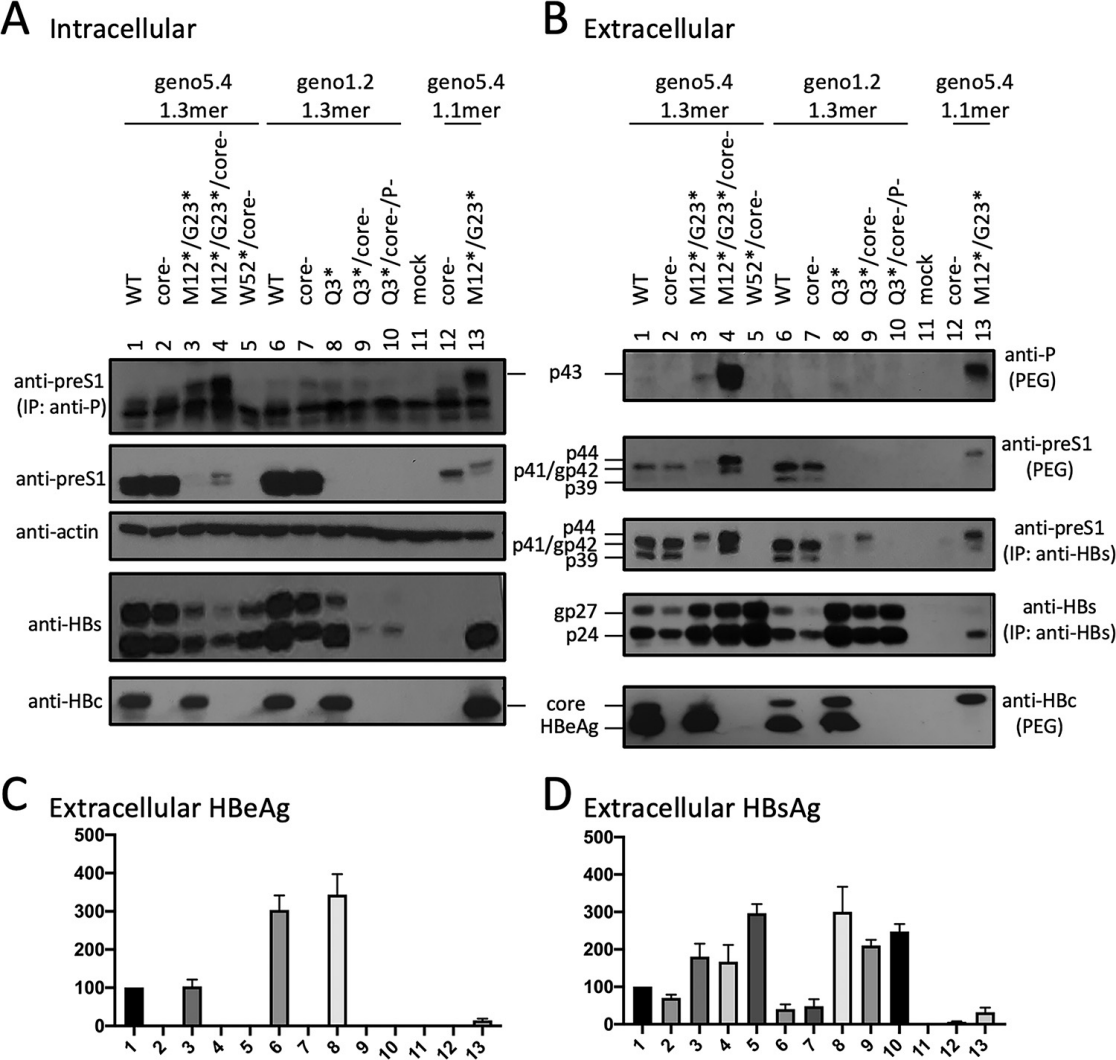
Production of p41/p44 from Huh7 cells transfected with 1.3-mer construct of some L-minus mutants of geno5.4 or geno1.2. Huh7 cells seeded in 6-well plates were transfected with 2 μg of indicated geno5.4 or geno1.2 clones as 1.3-mer construct or 1.1-mer construct, and harvested 4 days later. (A) Western blot analysis of cell lysate using anti-preS1 (2nd panel), anti-HBs (4th panel), or anti-HBc antibody (bottom panel), using β-actin as a loading control (3rd panel). Alternatively, one-half of the cell lysate was subjected to IP with the anti-P antibody followed by Western blotting with anti-preS1 antibody (top panel). (B) Western blot analysis of HBV proteins in culture supernatant following PEG precipitation (top, 2nd, and bottom panels) or IP with anti-HBs antibody (3rd and 4th panels). 45E9 was used as the anti-HBs antibody for Western blot analysis of both cell lysate and culture supernatant. (C and D) ELISA of secreted HBeAg (C) and HBsAg (D), with values for WT geno5.4 set at 100.

The 1.1-mer construct used is artificial in its overproduction of pgRNA by the strong cytomegalovirus (CMV) promoter. We therefore converted some 1.1-mer constructs to 1.3-mer constructs by deleting the CMV promoter from the vector and extending the 5′ end of the HBV insert. When transfected into Huh7 cells, the 1.3-mer constructs released more HBsAg than corresponding 1.1-mer constructs (Fig. 12D, compare lanes 2 and 3 with lanes 12 and 13). They also secreted HBeAg, which was abolished by the C48* core-minus mutation (Fig. 12C). Western blot analysis of culture supernatant revealed 17-kDa HBeAg in addition to the 21-kDa core protein, with slower migration of genotype A-derived HBeAg as anticipated (Fig. 12B, bottom panel) (34). Strikingly, p41/p44 could be detected in culture supernatant from the Q3* mutant of geno1.2 as a 1.3-mer construct, which was enhanced by the core-minus mutation (Fig. 12B, third panel, lanes 8 and 9). A higher extracellular level of p41/p44 was produced by the M12*/G23* mutant of geno5.4, which when combined with the core-minus mutation became comparable in intensity to L protein from the parental construct (Fig. 12B, third panel, compare lanes 1, 3, 4). Besides the 7H11 anti-preS1 MAb, extracellular p41/p44 from the 1.3-mer construct could also be detected by the 2C8 anti-P MAb following polyethylene glycol (PEG) precipitation (Fig. 12B, top). In cell lysate, a low level of p41/p44 was detectable from the M12*/G23* mutant of geno5.4, which was increased by a core-minus mutation (Fig. 12A, second panel, lanes 3 and 4). IP with anti-P MAb followed by Western blotting with anti-preS1 MAb provided more sensitive p43 detection, which revealed a low level of intracellular p43 from the 1.3-mer construct of geno1.2 as well (Fig. 12A, top, lanes 6 to 9).

### Efficient secretion of p41/p44 from L-minus mutants could be reversed by providing L/M proteins in *trans*.

**FIG 13:**
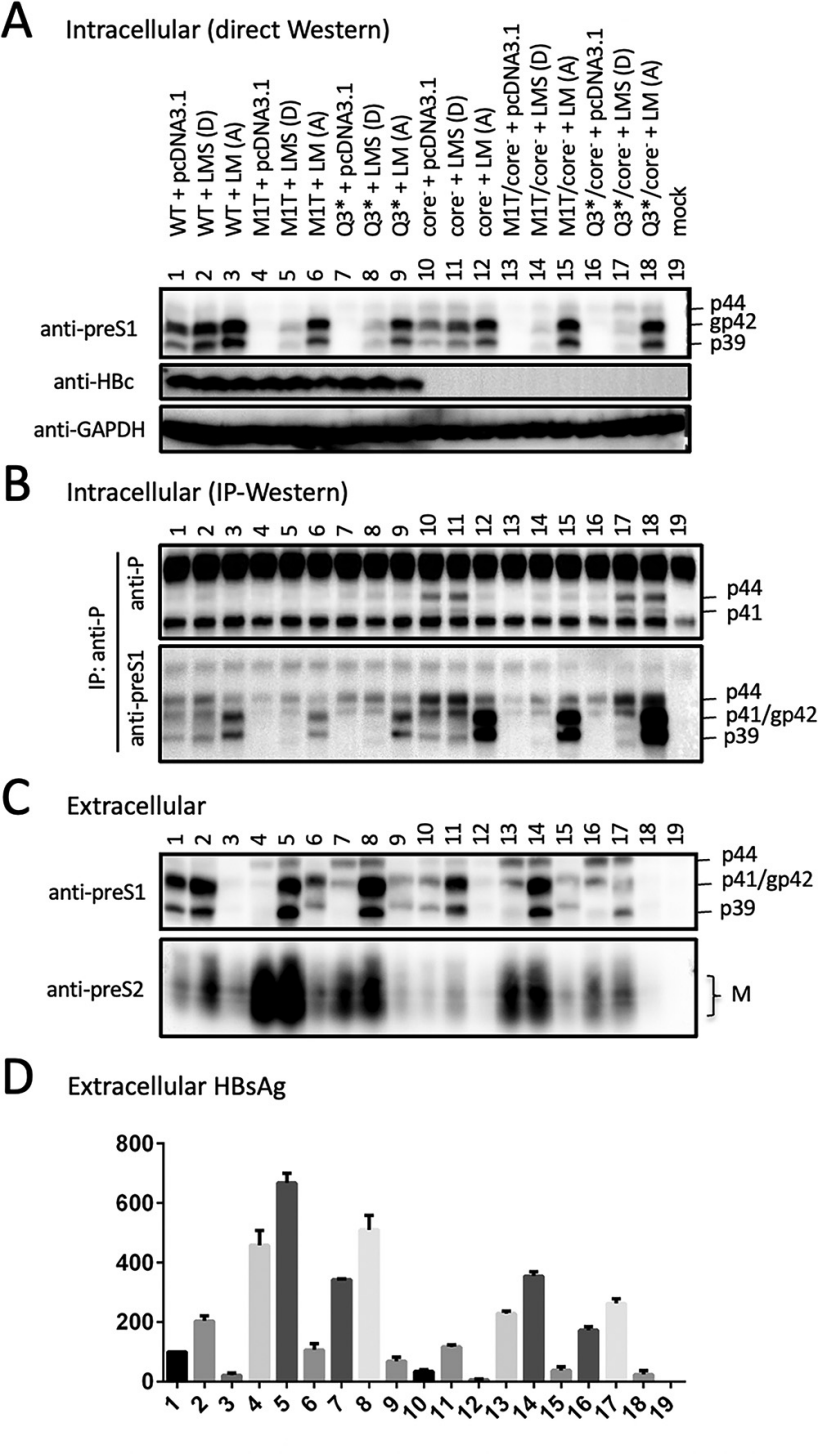
Intracellular and extracellular levels of p41/p44 from L-minus 1.1-mer constructs of genotype D following cotransfection with 0.7-mer L/M/S or L/M expression construct. Huh7 cells seeded in 6-well plates were transfected with 1.5 μg of 1.1-mer geno1.2 construct with or without core-minus and/or L-minus (M1T or Q3*) mutation, together with 0.5 μg of pcDNA3.1 vector, 0.1 μg of 0.7-mer L/M/S expression construct of genotype D [LMS (D)] plus 0.4 μg of pcDNA3.1, or 0.5 μg of 0.7-mer L/M expression construct of genotype A [LM (A)]. (A) Direct Western blot analysis of intracellular L and core proteins using GAPDH as a loading control. (B) IP-Western blot analysis of intracellular L protein and P-L fusion protein. One-third of the cell lysate was subjected to IP with 2C8, the anti-P MAb followed by sequential Western blot with 2C8 and 7H11, the anti-preS1 MAb. (C) Western blot analysis of secreted L and M proteins following PEG precipitation from culture supernatant. (D) ELISA of secreted HBsAg, with the value for WT 1.1-mer construct cotransfected with pcDNA3.1vector set at 100.

Taken together, the data presented so far suggested that the loss of L protein expression unleashed p41/p44 secretion from both genotype A and genotype D. To validate an inhibitory effect of L protein on p43 secretion, we cotransfected Huh7 cells with 1.5 μg of a 1.1-mer genotype D construct and 0.5 μg of pcDNA3.1 vector or N67, a 0.7-mer L/M expression construct of genotype A (19). Alternatively, the 1.1-mer construct was cotransfected with 0.1 μg of the 0.7-mer L/M/S expression construct of genotype D together with 0.4 μg of pcDNA3.1 vector. As expected, cotransfection with the 0.7-mer L/M construct suppressed M protein and HBsAg secretion from all six 1.1-mer constructs (Fig. 13C, bottom, and Fig. 13D). L protein became detectable inside cells cotransfected with L-minus (M1T or Q3*) 1.1-mer constructs and increased following cotransfection with the WT construct and its core-minus mutant (Fig. 13A, top), although extracellular L protein was rather reduced for the WT construct and its core-minus mutant (Fig. 13C, top). For all four L-minus mutants as 1.1-mer constructs, cotransfection with 0.7-mer L/M construct markedly reduced p44 in culture supernatant (Fig. 13C, top, lanes 4 versus 6, 7 versus 9, 13 versus 15, and 16 versus 18). This was accompanied by increased intracellular p44 for the L/core double-minus mutants (Fig. 13A, top, compare lanes 13 and 15 and lanes 16 and 18). IP with the 2C8 anti-P MAb followed by Western blot analysis confirmed increased intracellular p41/p44 for these two double mutants but not for the M1T or Q3* single mutant or for the core-minus mutant (Fig. 13B). Cotransfection with a small amount of the 0.7-mer L/M/S construct of genotype D increased intracellular p41/p44 for the M1T/core-minus and Q3*/core-minus double mutants without significantly reducing the extracellular level (Fig. 13B and C, top, compare lanes 13 and 14 and lanes 16 and 17).

## DISCUSSION

Four size forms of unspliced HBV RNAs generate 7 viral proteins. However, RNA splicing has the potential to generate fusion proteins between different genes (reviewed in references 35–37). A 2.2-kb spliced RNA was first detected from human liver samples and Huh7 and HepG2 cell lines transfected with a genotype D clone (38–40). Mapping of its 5′ end suggested that it could originate from either pcRNA or pgRNA (39). The 2.2-kb RNA contained a deletion of 1,223 bp (not a multiple of three) to join nt 2447 near the end of core gene with nt 489 in the middle of the S region (Table 2). The deletion converts P protein into a fusion polypeptide between its N-terminal 47 aa and a new peptide of 13 to 64 aa (depending on the HBV genotype) encoded by another reading frame in the S gene, which has been called HBV splicing-generated protein (HBSP). Second, it creates a new stop codon in the core gene to delete the last residue in the core protein (Table 2). Third, it creates a new ATG codon to express just the C-terminal portion of P protein of 377 aa. With the help of full-length P protein produced by pgRNA, the 2.2-kb spliced pgRNA can be packaged and reverse transcribed to DNA (41, 42). This splicing has been independently confirmed and can be detected in transgenic mice of a non-D HBV genotype (43, 44). In fact, it is the most common splicing variant of the 3.5-kb RNA (45).

**TABLE 2:** Impact of pgRNA splicing on coding capacity for core and P proteins^*a*^

RNA size (kb)	No. of nt deleted	RNA junction	Impact on core gene product	Impact on P gene product
3.5 (unspliced)	0	NA	183 aa	832 aa
2.2	1,223	nt 2447-nt 489	182 aa (last residue lost)	fusion: P – another ORF (**47 aa + 13 aa**)^*b*^; translation of C-terminal P (**377 aa; 42 kDa**)
2.7	685	nt 2985-nt 489	183 aa	fusion: P-S (227 aa + 115 aa)
3.0	454	nt 2447-nt 2902	fusion: core-P (183 aa + 633 aa)	fusion: P-L (**47 aa + 371 aa**)
2.1 (doubly spliced)	282 + 1,016	nt 2067-nt 2350; nt 2447-nt 282	94-aa internal deletion; fusion: core-S (89 aa + 183 aa)	translation of C-terminal P (436 aa)

a Based on an HBV DNA clone of genotype D (66). Underlining indicates deletions that are not in frame (multiples of three). Bold indicates proteins that have been proven experimentally.

b Known as HBSP.

A singly spliced RNA of 2.7 kb also has the 3′ junction at nt 489 but a different 5′ junction at nt 2985 (46). This 685-nt deletion will generate a fusion protein containing the N-terminal 227 aa of P protein and C-terminal 115 aa of S protein (Table 2). A doubly spliced 2.1-kb RNA has the nt 2067-nt 2350 and nt 2447-282 junctions (43, 47). The first splicing would make a 94-aa internal deletion in the core protein, while the second one would join that shortened (89-aa) core protein with the C-terminal 183 aa of S protein (Table 2). Moreover, it generates a new ATG codon with the potential to express another version of C-terminal P protein. By sequencing truncated HBV DNA inside virions from patient blood, Gunther et al. identified nt 2447 as the most common splice donor site, followed by nt 2067, nt 2985, nt 2087, and nt 2471 (nomenclature based on genotype D) (45). The splice acceptor sites were, from the most abundant, nt 489, nt 282, nt 2350, and nt 2902. They also identified a new doubly spliced transcript of 2.3 kb with nt 2447-nt 2902 and nt 2985-nt 489 junctions. That approach has the bias of selecting for those capable of encapsidation and conversion to progeny DNA.

From a completely different perspective, coding sequence for a protein kinase A (PKA) site was introduced into the 5′ end of P gene to permit *in vitro* P protein labeling by ^32^P (28). This enhanced detection sensitivity by 2 orders of magnitude when combined with IP with an antibody against the N terminus of P protein. Surprisingly, the P gene expression construct of genotype D generated large amount of a 43-kDa protein in addition to the full-length P protein of 90 kDa. p43 was associated with nucleocapsids just like full-length P protein, although it was highly susceptible to proteinase K digestion, arguing for its binding to nucleocapsids from outside (28). Subsequently, Huang and colleagues found that p43 could also be produced from a 1.1-mer construct of the genotype D clone (29). It could not be immunoprecipitated by an antibody against the C terminus of P protein and was eliminated by moving the PKA site to the 3′ end of P gene. Further study suggested that p43 was a glycoprotein containing preS1, preS2, and S epitopes. RT-PCR identified RNA splicing joining nt 2447 (third position at codon 47 of P gene) with nt 2902 (first position at codon 19 of preS1 region), with or without additional nt 2985-nt 489 splicing, as reported elsewhere (45). The single splicing of 454 nt with the nt 2447-nt 2902 junction (would be 487 nt for non-D genotypes) joins the N-terminal 47 aa of P protein with the C-terminal 371 aa of L protein (Table 2). It also fuses the entire core gene with the P gene downstream to generate a core-P fusion protein of 183 aa + 632 aa (Fig. 1C), which remains to be verified experimentally. An A2900C mutation at the conserved splicing acceptor site, while silent in the P gene, eliminated p43 production (29).

We previously found that S protein coexpression is required to sustain both intracellular and extracellular levels of M protein (14). Lost S protein expression from a 0.7-mer L/M/S expression construct or a 1.1-mer replication construct also reduced the intracellular level of L protein despite its blocked secretion (19). Moreover, a C48* nonsense mutation in the core gene of the 1.1-mer construct reduced all three envelope proteins in both cell lysate and culture supernatant, thus suggesting a role of core protein in stabilizing envelope proteins. To study this further, we would like to generate an L-minus mutant for the 1.1-mer construct of geno5.4, a genotype A clone, and geno1.2, a genotype D clone. However, as described here, mutating the preS1 ATG codon(s) or introducing a nonsense mutation immediately downstream caused a slight upshift of the extracellular p39/gp42 doublet to about 41/44 kDa (Fig. 2B and 3B). Western blot analysis revealed both preS1 (7H11) and preS2 (3E6) epitopes on p41/p44, while tunicamycin treatment established p44 as the glycosylated form of p41 (Fig. 6B and 7B).

Three pieces of evidence validated p41/p44 as p43, the previously reported P-L fusion protein (28, 29): the ability of an antibody against the N terminus of P protein (2C8) to recognize p41/p44 in IP and Western blot analysis (Fig. 5, 9, 10, and 13), the ability of a nonsense mutation at the 5′ P gene (L13*) to prevent p41/p44 expression (Fig. 9), and the ability of the A2900C mutation to eliminate p41/p44 production (Fig. 8). Revelation of p41/p44 as p43 could explain the similar mobility of p41/p44 between genotypes A and D despite faster migration of L protein from geno1.2 than geno5.4 (Fig. 3B). The predicted size of p43 is 418 aa (47 aa + 371 aa) for genotype D and 420 aa (49 aa + 371 aa) for genotype A. The 11-aa size difference in L protein is lost in p43 (Fig. 1B). The 7H11 epitope is retained in p43 (Fig. 1B), thus enabling its detection of p41/p44. The low preS1 signal according to ELISA (Fig. 2C, 3C, and 9E) is most likely attributed to a (nearly) lost epitope. The predicted p43 coding sequence could also explain why p41/p44 was eliminated by more downstream nonsense mutations (W52* in geno5.4, K38* in geno1.2). Translation of p43 from a derivative of 3.5-kb RNA rather than 2.4-kb RNA could explain why p41/p44 was not generated by the M1* mutation in the 0.7-mer L/M/S expression construct of geno1.2 (Zhang et al., unpublished).

Most chimeric proteins encoded by spliced RNA have not been validated by Western blotting (Table 2). Although single splicing of the 3.5-kb RNA to generate the nt 2447-nt 489 junction is the most common (45), HBSP expression has not been demonstrated in cells transfected with the 1.1-mer HBV DNA construct. Only one report described HBSP in liver samples from HBV infection (48), although its antibody or T cell immune response could be demonstrated for some chronic carriers (48, 49). The 42-kDa N-terminally truncated P protein, another product of that splicing, was indeed expressed in transfected cells but at a level similar to that of the full-length P protein (50). P protein is expressed at extremely low levels, as only one molecule is packaged into a core particle assembled from 240 copies of core protein to drive genome replication. It is preferentially translated from pgRNA rather than pcRNA (51, 52). Both HBSP and p43 share with P protein the N-terminal 47 aa (49 aa for genotype A), and hence their translation initiation would cover a similar 0.5-kb upstream sequence (if by ribosomal leaky scanning from the 5′ end). Whether HBSP and p43 are translated preferentially from spliced pgRNA or spliced pcRNA remains to be established, but our 1.1-mer construct should produce pgRNA but no pcRNA. Although single splicing to generate an nt 2447-nt 2902 junction seems uncommon according to analysis of virion DNA (45), previous studies found that p43 was associated with nucleocapsids at a level about 10 times higher than full-length P protein (28, 29). In this study, we found that many L-minus mutations in the 1.1-mer construct could render p41/p44 detectable in culture supernatant, which was further increased by the C48* core-minus mutation to approach the level of L protein from the parental construct (Fig. 3B, 5B, and 8B). Most strikingly, the ability of some L/core double minus mutations to dramatically increase p43 protein level could be reproduced from 1.3-mer construct (Fig. 12), which produces physiological levels of pcRNA and pgRNA through the endogenous core promoter.

Did the loss of L protein expression increase p43 expression or reduce its degradation? The L-minus mutation in the 1.1-mer construct of geno1.2 did not markedly increase the spliced 3.0-kb RNA for p43 (Fig. 8C). L protein blocks S protein secretion according to the L/S protein ratio (8–10). It probably can also retain p43 to promote its intracellular degradation. Indeed, cotransfection with N67 eliminated extracellular p41/p44 from the L-minus mutants of geno1.2 (Fig. 13C). Certainly, N67 also produces M protein and a small amount of an N-terminally truncated S protein, which is secretion deficient and could inhibit secretion of the full-length S protein (19). p43 lacks the N-terminal 18 aa (genotype D) or 29 aa (genotype A) of the preS1 domain and is no longer myristoylated due to a proline at position 2 (Fig. 1A). In this regard, an E2G mutation in the S protein to confer myristoylation markedly inhibited its secretion (53–55), although preventing myristoylation of L protein by mutating the glycine residue(s) may or may not promote its secretion depending on HBV genotype (53, 56). For genotype A, preS1 aa 6 to 19 were found to be responsible for intracellular retention of L protein and its inhibition of S protein secretion (56). Indeed, deleting the N-terminal 22 aa or 30 aa from the preS1 domain of genotype A enabled L protein secretion, even at a high L/S protein ratio, and abolished its inhibition of S protein secretion (6, 57). Therefore, loss of the N-terminal preS1 sequence in p43 most likely confers a propensity for efficient secretion, which for WT HBV is blocked by L protein coexpression.

While the C48* core-minus mutation reduced both intracellular and extracellular levels of L, M, and S proteins for the WT construct and M and S proteins for the L-minus mutants, it further increased p41/p44 for all the L-minus mutants in culture supernatant, whether for the 1.1-mer construct or the 1.3-mer construct (Fig. 2B, 3B, 4A and B, 5B, 8B, 10B, and 12B). It also increased p41/p44 in cell lysate for the G23* and M12*/G23* L-minus mutants of genotype A (as 1.1-mer or 1.3-mer constructs) but only intracellular p41/p44 for the WT genotype D construct as a 1.1-mer construct (Fig. 3A, 5A, 10A, and 12A). Thus, the C48* mutation increased p43 protein level but did not promote its secretion. Two hypotheses, not necessarily mutually exclusive, could explain such findings. First, the C48* nonsense mutation, which is located upstream of the P gene in the 3.0-kb spliced RNA, promotes translational termination and reinitiation at the P gene AUG to augment p43 expression. Second, intracellular core particles interact with p43 in addition to L, M, and S proteins, which stabilizes the envelope proteins but destabilizes p43. p43 was discovered through IP of core particles by anti-HBc antibody followed by denaturation, re-IP by the anti-P antibody, and phosphorylation (28, 29). In support of the destabilizing effect of core protein (or the presumptive core-P fusion protein), blocking p43 secretion from the L-minus mutants by N67, the 0.7-mer L/M construct, led to increased intracellular level of p41/p44 for the M1T/core-minus and Q3*/core-minus double mutants but not for the M1T or Q3* single mutant (Fig. 13B and C). More studies are needed to dissect the translational versus stabilizing effect of the C48* core-minus mutation.

The present study demonstrated ability of L-minus mutants to secrete large amounts of p43 from either the 1.1-mer or 1.3-mer HBV DNA construct. To check for p43 expression from authentic cccDNA of such mutants requires infection experiments in HepG2/NTCP cells or differentiated HepaRG cells (58, 59), using viral inoculum generated by cotransfection between the L-minus 1.1-mer construct and an L protein expression construct. A major unanswered question is that of the p43 protein level during natural infection by WT HBV, its distribution on virions versus SVPs, and the functional consequence of its loss of expression. We detected small amounts of p43 from lysates of Huh7 cells transfected with 1.1-mer (and 1.3-mer) WT constructs of geno1.2 but not from culture supernatants of cells transfected with WT HBV of either geno1.2 or geno5.4. Still, it remains possible that secreted p43 from WT HBV is enriched on virions rather than SVPs. p43 retains the matrix domain at the preS1/preS2 junction required for capsid interaction (6), and previous work found its association with secreted virions and SVPs (29). Since myristoylated preS1 peptide 2–18 (genotype D) or 2–29 (genotype A) lost in p43 constitutes the minimum binding site for the high-affinity HBV receptor (60, 61), p43 incorporated into virions will downregulate HBV infectivity. Even if the steady-state level of p43 is low on secreted virions, dynamic p43 interaction with L and core proteins could modulate virion formation or release or genome maturity. Therefore, it will be important to examine the functional consequences of mutations to abolish p43 expression from 1.1-mer and 1.3-mer HBV DNA constructs. Whether overexpression of p43 or its variant forms can inhibit HBV infection in a dominant negative manner also warrants further investigation.

## MATERIALS AND METHODS

### 1.1-mer HBV DNA construct and site-directed mutagenesis.

The genotype A clone geno5.4 (GenBank accession number KX827293) and genotype D clone geno1.2 (GenBank accession number KX827290) were generated from PCR products of patient serum samples and available as SphI dimers (20). They were subsequently converted to 1.1-mer constructs with pgRNA transcription driven by the CMV promoter. This was achieved by inserting nt 1805 to 3221 and 1 to 1932 of geno5.4 and nt 1805 to 3182 and 1 to 1932 of geno1.2 into the SacI-HindIII sites of the pcDNA3.1 Zeo^−^ vector (19). The core-minus mutant (C48*) and S-minus mutant (M1T) in the 1.1-mer construct have been previously described, as were the L-minus mutants W52* for geno5.4 and K38* for geno1.2 (Table 1) (19). Additional L-minus mutants were generated by overlap extension PCR, with the specific mutations and impact on P protein translation shown in Table 1. The P-minus mutation (L13*) in geno1.2 was achieved by a T2344A change (from TTG to TAG), which was silent in the overlapping core gene. The A2900C mutation in genotype D has been shown by others to prevent splicing of 3.5-kb RNA needed to generate the nt 2447-nt 2902 junction (29).

### Conversion of the 1.1-mer HBV DNA construct to the 1.3-mer construct.

The 1.3-mer construct differed from the 1.1-mer construct by extending the 5′ end of HBV insert from nt 1805 to nt 1031 and by removing a circa 0.8-kb vector sequence upstream of the HBV insert including the CMV promoter. For geno5.4, the recently described approach was used (59). Briefly, a 1.0-kb HBV DNA fragment (nt 1031 to nt 2035) and an overlapping 0.6-kb fragment (nt 2013 to nt 2621) were generated by PCR using primer pairs p1S/p1As and p2S/p2As, respectively. The PCR products were gel purified and assembled with the 7-kb MfeI-ApaI restriction fragment of the original 1.1-mer construct using a NEBuilder Hifi DNA assembly cloning kit (New England Biolabs). For geno1.2, the 1.0-kb fragment (nt 1031 to nt 2035) and an overlapping 1.3-kb fragment (nt 2013 to nt 3182 and nt 1 to nt 151) were amplified using primer pairs p3S/p3As and p4S/p4As for assembly with the 6.2-kb NruI-XhoI fragment of the original 1.1-mer construct. The primer sequences are as follows: p1S, CAAGGCAAGGCTTGACCGACAATTGCACAATGTGGATATCCTGCCTTAATG (MfeI site underlined); p1As, AGGAGACTCTAAGGCTTCTCGATACAG; p2S, ATCGAGAAGCCTTAGAGTCTCCTGAGC; p2As, CTTCTCTTTTCATTTACAGTGAGAGGGCCCAC (ApaI site underlined); p3S, TTAGGCGTTTTGCGCTGCTTCGCGACACAATGTGGTTATCCTGCTTTAATGC (NruI site underlined); p3As, AGGAGACTCTAAGGCTTCCCGATAGA; p4S, CTCTATCGGGAAGCCTTAGAGTCTCCTGAGC; and p4As, CAGCGCAGGGTCCCCAGTCCTCG.

### 0.7-mer L/M/S expression construct and its S-minus mutant.

The 0.7-mer construct N51 of genotype A had a subgenomic fragment of the HBV genome cloned in the pBluescript SK(−) vector to permit expression of L, M, and S proteins under the control of endogenous SPI and SPII promoters (14, 19). N67 was derived from N51 by mutating the S gene ATG to GCG (M1A), thus preventing expression of full-length S protein (19). It could produce small amounts of N-terminally truncated S protein through translation initiation from the next in-frame ATG codon (M75). For the present study, a 0.7-mer L/M/S construct was made for geno1.2 of genotype D using the same DNA fragments as the 0.7-mer construct of genotype A.

### Transient transfection, Western blotting, immunoprecipitation, and ELISA.

The human hepatoma cell line Huh7 was cultured in Dulbecco’s modified Eagle’s medium supplemented with 10% fetal bovine serum and 100 U/ml penicillin and 100 μg/ml streptomycin. HepG2 cells were cultured in minimum essential medium (MEM) supplemented with 10% fetal bovine serum, MEM nonessential amino acids solution, 100 U/ml penicillin, and 100 μg/ml streptomycin. Cells seeded in 6-well plates were transfected with 2 μg of DNA construct using the Mirus reagent. Cells and culture supernatant were harvested 4 days posttransfection. Cells were lysed in buffer consisting of 10 mM HEPES, pH 7.5; 100 mM NaCl; 1 mM EDTA; 1% NP-40; and cOmplete protease inhibitor cocktail (Roche). The cell lysate (1/12 of total volume) was used for Western blot analysis of envelope and core proteins using established methods and reagents (19, 20, 58, 59, 62–64). 7H11 is a mouse monoclonal antibody (MAb) raised against preS1 aa 21 to 47 of a non-D HBV genotype (21, 22), and fine mapping revealed its epitope inside aa 34 to 47 (Q. Yuan, unpublished data). MAb 3E6 was raised against preS2 aa 6 to 27 of genotype D (26), while the anti-HBc MAb 2A7 recognizes aa 141 to 154 in core protein (65). S protein was detected in Western blots by the rabbit polyclonal anti-S antibody (Novus) or by mouse MAb 45E9 raised against aa 27 to 79 in S protein (65). 2C8, the anti-P MAb that works in Western blotting, IP, and immunofluorescent staining (30–32), recognizes aa 8 to 20 in HBV P protein (sc-81590; Santa Cruz). The dilutions of primary antibodies for Western blotting were 1:4,000 (7H11), 1:2,000 (3E6), 1:3,000 (Novus anti-S), 1:2,000 (45E9), 1:500 (2C8), and 1:10,000 (2A7). Secreted envelope proteins were precipitated from 120 μl of culture supernatant by 10% polyethylene glycol (PEG) prior to Western blotting. Alternatively, they were immunoprecipitated from 250 μl to 600 μl of culture supernatant by overnight incubation at 4°C with 3E6 (1:500 dilution), horse anti-Ad/Ay antibody (Abcam; 1:500 dilution) (58), or 2C8 (1:100 dilution) conjugated to protein G-Sepharose beads. For IP of cell lysate, one-third or one-half of the cell lysate was incubated overnight in 100 μl volume with anti-Ad/Ay antibody (1:20 dilution) or 2C8 MAb (1:12 dilution). Secreted HBsAg and HBeAg were quantified by enzyme linked immunosorbent assays (ELISA) using commercial kits (Kehua, Shanghai, China), with proper sample dilution to avoid signal saturation. The preS1 antigen was detected from undiluted culture supernatant using an ELISA kit for hepatitis B virus preS1 antigen (Kehua).

### Tunicamycin treatment of transfected cells.

Huh7 cells seeded in 6-well plates were transfected in triplicate with different 1.1-mer constructs. About 57 h, later one well was treated with 1% DMSO and another well with 50 μg/ml of tunicamycin dissolved in 1% DMSO. The third well was left untreated. Cells and culture supernatant were harvested 42 h later for Western blot analysis of envelope and core proteins.

### PCR detection of spliced 3.5-kb RNA with the nt 2447-nt 2902 junction.

Huh7 cells seeded in 12-well plates were transfected with 1 μg of 1.1-mer HBV DNA construct using Lipofectamine 3000 transfection reagent (Invitrogen). Cells were lysed 72 h later with TRI reagent (MRC). The lysate was mixed with equal volume of chloroform, and after centrifugation, RNA in the aqueous phase was precipitated with isopropanol. The RNA pellet was washed with 70% ethanol and absolute ethanol and dissolved in diethyl pyrocarbonate (DEPC)-treated water. RNA (2 μg) was reverse transcribed to cDNA using a PrimeScript II First Strand cDNA synthesis kit (TaKaRa) in a total volume of 20 μl. The cDNA (3 μl) was used for PCR amplification of cDNA derived from 3.5-kb HBV RNA using forward primer P1 (nt 2310 to 2329; CCCCTATCTTATCAACACTT) and reverse primer P2 (nt 139 to 120; CCCAGTCCTCGAGAAGATTG), similar to that described in previous work on p43 (29). The PCR conditions were initial denaturation at 98°C for 60s, followed by 30 cycles of 98°C for 15 s, 63°C for 15 s, and 72°C for 30 s, and a final extension at 72°C for 5 min. PCR products were separated on 1.3% agarose gels. The 1.2-kb, 1.0-kb, 0.85-kb, and 0.56-kb bands were cut out and purified with a DNA gel extraction kit (Axygen). The Mighty TA-Cloning reagent set for PrimeSTAR (TaKaRa) was used to add an A tail to the PCR products for their ligation to the pMD20-T vector. Clones with insert were identified by blue-white color screening, and individual recombinant clones were sequenced using universal primers from the vector: M13R (CAGGAAACAGCTATGAC) and M13F (CGCCAGGGTTTTCCCAGTCACGAC).

### Statistical analysis.

Differences between the groups were examined by using Student's *t* test. *P* values of <0.05 were considered statistically significant. All experiments were repeated 3 times, and data are presented as means and standard deviations (SD).
